# Chronotype and environmental light exposure in a student population

**DOI:** 10.1080/07420528.2018.1482556

**Published:** 2018-06-18

**Authors:** Kate Porcheret, Lucien Wald, Lin Fritschi, Menno Gerkema, Marijke Gordijn, Martha Merrrow, Shantha M.W. Rajaratnam, Daniel Rock, Tracey L. Sletten, Guy Warman, Katharina Wulff, Till Roenneberg, Russell G. Foster

**Affiliations:** a Sleep and Circadian Neuroscience Institute (SCNi), Nuffield Department of Clinical Neurosciences, University of Oxford, Oxford, UK; b Centre for Observation, Impacts, Energy, MINES ParisTech, PSL Research University, Paris, France; c School of Public Health, Curtin University, Perth, Australia; d Chronobiology unit, Groningen Institute for Evolutionary Life Sciences, University of Groningen, Groningen, The Netherlands; e Chrono@Work, Groningen, The Netherlands; f Molecular Chronobiology, Institute of Medical Psychology, LMU Munich, Munich, Germany; g Monash Institute of Cognitive and Clinical Neurosciences and School of Psychological Sciences, Monash University, Melbourne, Australia; h WA Primary Health Alliance, Perth, Australia; i School of Psychiatry and Clinical Neurosciences, The University of Western Australia, Perth, Australia; j Chronobiology Group, Department of Anaesthesiology, School of Medicine, Faculty of Medical and Health Sciences, University of Auckland, Auckland, New Zealand

**Keywords:** Chronotype, Light, MCTQ, Students, Daily irradiance

## Abstract

In humans and most other species, changes in the intensity and duration of light provide a critical set of signals for the synchronisation of the circadian system to the astronomical day. The timing of activity within the 24 h day defines an individual’s chronotype, i.e. morning, intermediate or evening type. The aim of this study was to investigate the associations between environmental light exposure, due to geographical location, on the chronotype of university students. Over 6 000 university students from cities in the Northern Hemisphere (Oxford, Munich and Groningen) and Southern Hemisphere (Perth, Melbourne and Auckland) completed the Munich ChronoType Questionnaire. In parallel, light measures (daily irradiance, timing of sunrise and sunset) were compiled from satellite or ground stations at each of these locations. Our data shows that later mid-sleep point on free days (corrected for oversleep on weekends MFS_sc_) is associated with (i) residing further from the equator, (ii) a later sunset, (iii) spending more time outside and (iv) waking from sleep significantly after sunrise. However, surprisingly, MSF_sc_ did not correlate with daily light intensity at the different geographical locations. Although these findings appear to contradict earlier studies suggesting that in the wider population increased light exposure is associated with an earlier chronotype, our findings are derived exclusively from a student population aged between 17 and 26 years. We therefore suggest that the age and occupation of our population increase the likelihood that these individuals will experience relatively little light exposure in the morning whilst encountering more light exposure later in the day, when light has a delaying effect upon the circadian system.

## Introduction

The circadian system adjusts physiology and behaviour to the varied demands of the day–night cycle (Czeisler et al. 1999; Wright et al. 2001; Roenneberg et al. 2003a). To ensure synchrony with the astronomical day, the circadian system entrains to daily environmental signals (zeitgebers = time givers). The light–dark cycle is the most significant zeitgeber for most organisms, including humans (Honma et al. 1987; Roenneberg et al. 2007). Differences in the relationships between an individual’s circadian phase and external local time give rise to a distribution of chronotypes across the population, ranging from *early* chronotypes, the proverbial “larks”, to *late* chronotypes termed “owls” (Roenneberg et al. 2003a).

The timing of light exposure has a differential effect upon circadian phase: early light exposure advances the cycle whilst light late in the internal day delays circadian phase (Czeisler et al. 1989; Khalsa et al. 2003). Thus, exposure to bright artificial light in the evening before bedtime has been associated with a delay in circadian phase, as assessed by measures of subjective chronotype (Martin et al. 2012; Vollmer et al. 2012), subjective sleep timing (Koo et al. 2016), salivary melatonin levels (Gordijn et al. 1999; Benloucif et al. 2008; Cajochen et al. 2011) and core body temperature (Krauchi et al. 1997). Furthermore, adolescents living in urban areas and exposed to bright artificial light at night have a later chronotype as assessed by the Munich ChronoType Questionnaire (MCTQ) and Morningness–Eveningness Questionnaire (MEQ), compared to those living in more rural settings (Vollmer et al. 2012). By contrast exposure to bright light in the morning results in an advance of the circadian phase of melatonin synthesis and release (Dijk et al. 1989; Gordijn et al. 1999; Revell et al. 2005). In addition, bright morning light has been used as a therapy for advancing sleep timings in patients with delayed sleep–wake phase disorders (Rosenthal et al. 1990; Saxvig et al. 2014) and, more recently, with social jet lag (SJL) (Geerdink et al. 2016).

Despite society’s increasing detachment from the natural light–dark cycle, sunlight can still be seen to impact chronotype. Living further east within the same time zone in the Northern Hemisphere is associated with an earlier subjective chronotype in adults assessed using the MCTQ (Roenneberg et al. 2007) and in adolescents assessed with the MEQ (Randler 2008), most likely as a result of an earlier sunrise time. Seasonal changes are also apparent, such that during the months of increasing day length, subjective chronotype advances with individuals rising earlier (Kantermann et al. 2007; Allebrandt et al. 2014). There is also some evidence that geographical location has an impact upon chronotype. For example, in a study conducted in Brazil, subjective chronotype was assessed using the MCTQ and MEQ in two cities: São Paulo at latitude 23° 32' S and longitude 46° 38' W and Natal at 05° 47' S and 35° 12' W. Chronotype was found to be earlier in individuals living in Natal, the city closest to the equator (Miguel et al. 2014).

Clearly, the pattern of natural light within a particular environment will be critical in defining an individual’s phase of entrainment. However, an individual’s behaviour within that environment will also play an important role. A recent study compared the same individuals living under their normal urban routines (including artificial light at night) with a period under natural light exposure (camping without artificial light). The findings demonstrated that increased exposure to natural light advanced the circadian phase of all individuals (Wright et al. 2013; Stothard et al. 2017). Increasing photic zeitgeber strength by spending more time outside has also been correlated with self-reported chronotype: the more time spent outside, the earlier the chronotype (Roenneberg and Merrow 2007; Roenneberg et al. 2015).

By studying populations across the Northern Hemisphere and Southern Hemisphere, specifically Oxford, Groningen, Munich, Perth, Melbourne and Auckland, we aimed to investigate the association between geographical location and chronotype and how different aspect(s) of environmental light (timing; length of time spent outside; intensity of light, sleep timings relative to sunrise and sunset) might influence chronotype.

## Materials and methods

Students were recruited from six universities: University of Oxford, UK (51° 45' N, 1° 15' W); University of Groningen, The Netherlands (53° 13' N, 6° 33' E); LMU, Munich, Germany (48° 8' N, 11° 34' E); University of Western Australia, Perth, Australia (31° 57' S, 115° 51' E); Monash University, Melbourne, Australia (37° 48' S, 144° 57' E) and University of Auckland, New Zealand (36° 50' S, 174° 44' E). Students were asked to complete the online version of the MCTQ twice, in May and October of 2010, to control for seasonal influences. Overall, 13 299 individuals completed the MCTQ online. Over half of the participants were excluded from the analysis (see section on ‘Data processing’). 6 441 students (mean age 21.5 ± 2.2 years, 67.5% female, see Table 1 for group demographics) were included in the analysis. Daily irradiance, sunrise and sunset times were obtained for May and October 2010. Ethical approval for this study was obtained from the local ethics committee for each university involved in the study.

**Table 1.** Average demographics, sleep timings and social jet lag for each city.

	Oxford (*n* = 302)	Groningen (*n* = 3050)	Munich (*n* = 1919)	Perth (*n* = 342)	Melbourne (*n* = 368)	Auckland (*n* = 460)
Age (years)	21.1 ± 2.09	21.54 ± 2.14	22.50 ± 1.93	18.96 ± 1.38	20.51 ± 1.90	20.00 ± 1.98
Gender: females (%)	161(53.3)	1996(65.4)	1401(73.0)	202(59.1)	271(73.6)	314(68.3)
Workdays (local time)						
Bedtime	00:42 ± 01:09	00:19 ± 01:07	00:02 ± 01:06	23:02 ± 01:30	00:12 ± 01:23	23:01 ± 01:10
Wake-up time	07:55 ± 00:53	08:02 ± 01:03	07:30 ± 01:01	07:28 ± 01:13	07:20 ± 01:04	07:00 ± 01:02
Free days (local time)						
Bedtime	01:30 ± 01:24	01:14 ± 01:22	01:09 ± 01:23	00:46 ± 01:39	00:59 ± 01:32	00:46 ± 01:27
Wake-up time	09:52 ± 01:20	09:53 ± 01:20	09:41 ± 01:23	09:23 ± 01:32	09:45 ± 01:32	09:10 ± 01:28
Social jet lag (hours)	1.44 ± 0.83	1.39 ± 0.91	1.66 ± 0.93	1.46 ± 0.92	1.65 ± 0.98	1.62 ± 0.95

Mean and standard deviation shown.

## Materials

### The Munich ChronoType Questionnaire

The online version of the MCTQ (Roenneberg et al. 2003b) was used in the native language of the country of each university. The MCTQ consists of questions concerning sleep timings for both workdays and free days separately, work time and time spent outside. The MCTQ has been validated against actigraphic recordings (Vetter et al. 2015) and melatonin rhythms (Kitamura et al. 2014). The MCTQ is used to calculate the MSF as the mid-point between sleep onset and sleep end. MSF was corrected for oversleep on free days (MSF_sc_: Mid Sleep point on Free days, Sleep Corrected) that occurs as a result of sleep debt [MSF_sc_ = MSF – (SDf – ((((nWD × SDw) + (7 − nWD)) × SDf)/7)), where SDf is the sleep duration of free days, SDw is the sleep duration of workdays and nWD is the number of workdays (Roenneberg et al. 2004). In cases where the numbers of workdays were missing, five workdays were assigned. SJL was also calculated from the MCTQ [SJL = MSF – MSD] where MSF is the mid-sleep point of free days and MSD the mid-sleep point of work days (Wittmann et al. 2006).

### Light data

*“Time spent outside”* was self-reported on the MCTQ. The weighted average of the number of hours given for free days and workdays was calculated using the number of workdays also reported on the MCTQ. If no workdays were given, five workdays were assigned. [time spent outside = ((time spent outside on workdays × number of workdays) + (time spent outside on free days × (7 − number of workdays)))/7].

*“Light dose”* is a measure of how much light individuals are exposed to over a given period of time. Here, we calculated the average hourly irradiance for the day for participants that completed the MCTQ and normalised this to the “time spent outdoors”, averaged for work and free days [light dose = daily irradiance/day length × time spent outside].

*“Day length”* for each day of the collection periods, for each city, was calculated using the world clock (http://www.timeanddate.com/worldclock).

*“Daily irradiances”* for the collection periods, for both May and October 2010, were obtained from three sources on an hourly basis. The data for Oxford, Groningen and Munich were provided by Dr Lucien Wald (MINES, ParisTech), obtained from Meteosat satellite images and converted to data maps of solar radiation using the Heliosat-2 method (Rigollier et al. 2004). The data for Perth and Melbourne were obtained from the Australian Bureau of Meteorology, again derived from satellite images processed by the Australian Bureau of Meteorology. Finally, the data for Auckland were obtained from the New Zealand Meteorological office based on readings from its ground station in Auckland. For all daily irradiance, the data represent light intensity experienced at ground level, taking into account weather conditions, either through processing of satellite data or as data taken at ground level.

## Data processing

As only 440 participants completed the questionnaire in both May and October (420 from Northern Hemisphere cities and 20 from Southern Hemisphere cities), longitudinal analysis was not performed and data for these participants were only included in the data analysis from the May collection period. Individuals were excluded if they were outside the age range (17–26 years); did not indicate they were currently living in any of the cities of interest; completed the questionnaire outside May or October 2010; or had reported working shifts during the past 3 months. Individuals were also excluded if they indicated using an alarm clock on free days (an exclusion criterion for chronotyping). For inter-hemispheric comparisons, months were assigned to season (Northern Hemisphere, May, and Southern Hemisphere, October, as spring and *vice versa* as autumn).

## Data analysis

Statistical analysis was performed using R version 3.0.1 (2013-05-16, Copyright 2013 The R Foundation for Statistical Computing). Linear mixed-effects models were fitted using the lme package for group and seasonal assessments of MSF_sc_ and light data. For categorical comparisons, linear models were referenced to Oxford for group comparisons, females for sex comparisons and spring for season comparisons. Since age and sex are known to influence chronotype, both of these were included as covariants when modelling MSF_sc_. Spearman’s rank correlation analysis was performed to assess associations between MSF_sc_ and light data.

## Results

On average, students in this sample reported the following habitual sleep-related times: bed time on workdays: 00:11 ± 01:10 (mean, SD) and nearly an hour later on free days: 01:09 ± 01:24; wake-up time was two hours later on free days (09:45 ± 01:23) compared to workdays (07:43 ± 01:06). A mean of 1.51 ± 0.93 h of SJL was reported. Table 1 details wake-up and bed times for work and free days at each city along with SJL. The sleep midpoint on free days (corrected for over sleep; MSF_sc_) was different between cities but not between seasons, and no city–season interactions were found (see suppl. data model 1). Hence, MSF_sc_ was collapsed across seasons.

**Figure 1.**
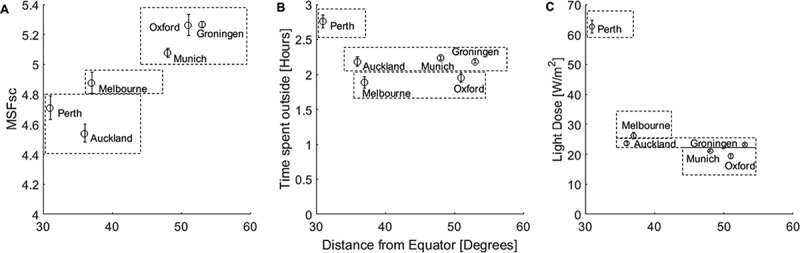
Relationship between mid-sleep time points (MSFsc) and ambient light conditions in students living in different cities of the Northern and Southern Hemispheres. Cities are plotted relative their distance to the equator and against (a) Average MSFsc (as time-of-day in hours (decimal time), (b) time spent outside and (c) light dose. Dashed lined boxes represent cities that are not statistically different to each other (see supplementary materials for models). Error bars = standard error of the mean. MSFsc: mid-sleep on free days sleep corrected.

When plotting, chronotype and time spent outside against the absolute distance of each city from the equator, MSF_sc_ showed a positive association: with chronotype becoming later with increasing distance (Figure 1a). Controlling for age and sex, the cities formed three groups for MSF_sc_: Oxford, Groningen and Munich were not statistically different from each other, and had the latest MSF_sc_; Melbourne showed an intermediate MSF_sc_ and was statistically significant from all the other cities; and Perth and Auckland showed the earliest MSF_sc_ and were also not statistically significant from each other (see suppl. data model 2).

Overall, the students (regardless of city) reported they spend on average (2.20 ± 1.33 h) outside a day resulting in exposure to on average (24.74 ± 20.09 W/m^2^) of light on the day they completed the survey (light dose). Students in Perth reported spending the most amount of time outside (2.76 ± 1.67 h) and experienced the highest intensity of light whilst outside, light dose (62.68 ± 39.11 W/m^2^, see suppl. data model 3 and 4). Whereas students in Melbourne reported spending the least amount of time outside (1.89 ± 1.49 h), students in Oxford received the lowest light dose (19.43 ± 14.31 W/m^2^). In relation to geographical location, the amount of time spent outside was not associated with the distance of each city from the equator (Figure 1b), but light dose did show an association, with the cities nearest to the equator experiencing a higher light dose, except for Auckland (Figure 1c).

**Figure 2.**
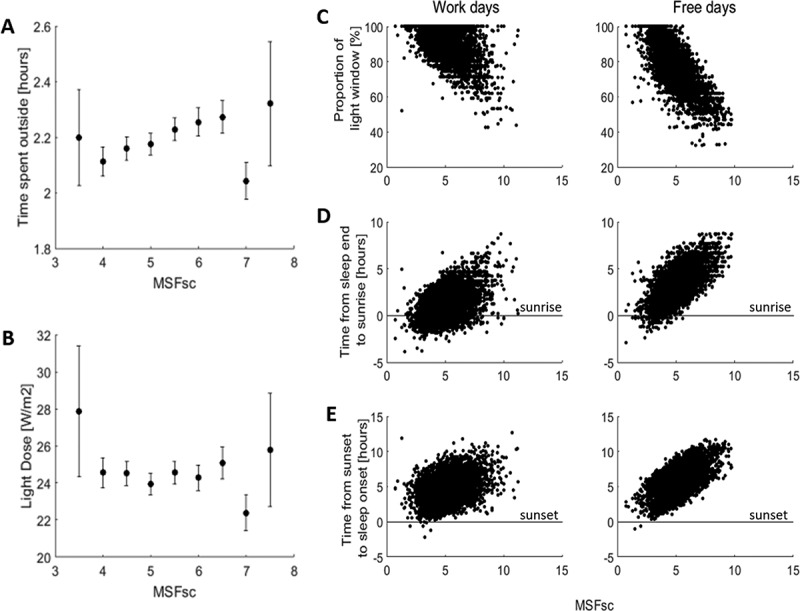
MSFsc and ambient light conditions. Average time spent outside (a) and light dose (b) for 30 minute bins of MSFsc across all students regardless of city. Error bars represent the standard error of the mean. (c) The shorter the proportion of the light window (time between sunrise and sunset) students are awake for, the later MSFsc for work and free days. (d) The later students wake up after sunrise (represented by line) the later MSFsc for work and free days. (e) The later student go to bed after sunset (represented by line) the later MSFsc for work and free days. MSFsc: mid-sleep on free days sleep corrected.

MSF_sc_ was positively, although weakly, correlated with time spent outside (rho = 0.036, *p* = 0.005) indicating that the longer students spent outside the later their sleep midpoint on free days. Time spent outside binned for MSF_sc_ in 30 min intervals, showed a stronger positive correlation (rho = 0.86, *p* = 0.011, Figure 2a). However, this was only statistically significant with the removal of the outlier of MSF_sc_ binned from 06:30 to 07:00. Light dose was not correlated with MSF_sc_ for raw (rho = − 0.0005, *p* = 0.97) or binned data (rho = − 0.15, *p* = 0.71, Figure 2b). Using a linear mixed effect model, taking age and sex into account, time spent outside but not light dose was found to have a significant effect on MSF_sc_ (see suppl. data model 5). However, the addition of time spent outside into the model did not remove the effect of city; moreover, no city-time spent outside interaction was found (see suppl. data model 6). This suggests that although the amount of time spent outside does have an influence on sleep midpoint on free days, other differences between the cities are also important.

To define the time of day, the students in this population were most likely to receive natural light, the timing of sunrise and sunset the day each student completed the survey was compared to their self-reported sleep timings for work and free days. The proportion of daylight (i.e. between sunrise and sunset) during which time students were awake was negatively correlated to MSF_sc_ for both work (rho = − 0.4, *p* < 0.001) and free days (rho = −0.71, *p* < 0.001, Figure 2c), indicating that students with the latest MSF_sc_ were only likely to be awake for around 40% of the daylight period. This is because students wake up after sunrise rather than going to bed before sunset (Figure 2d and 2e, respectively). About 75.5% of students woke up after sunrise on workdays and 98.1% on free days, with 15.7% waking up 5 h after sunrise on free days (1.2% on workdays).

**Figure 3.**
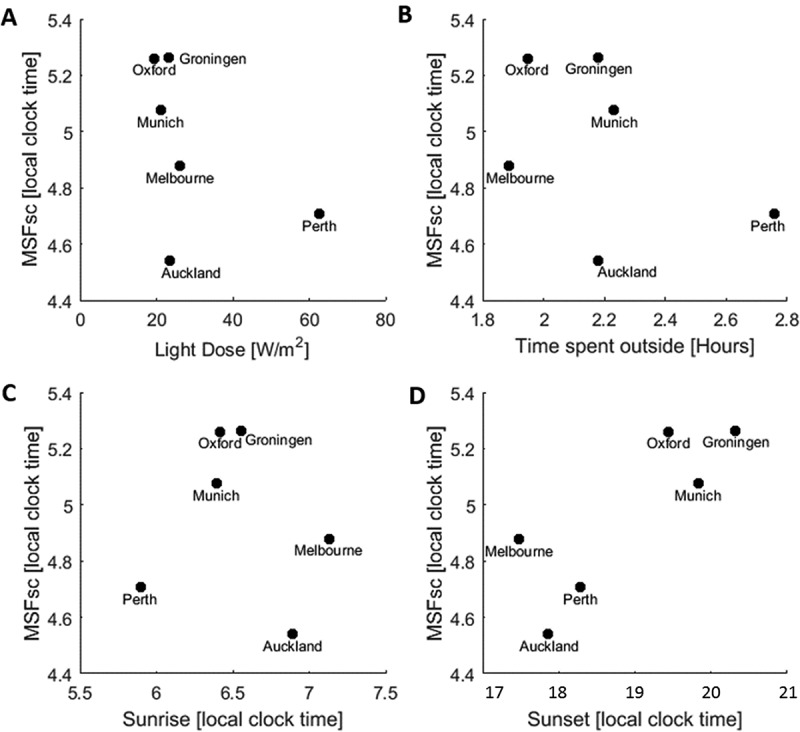
Geographical location, MSFsc and ambient light conditions. Average MSFsc for each city plotted against light dose (a), time spent outside (b), time of sunrise (c) and time of sunset (d). Sunset times given as hours from midnight.

The impact of geographical location on MSF_sc_ was found to be most influenced by the timing of sunset. The average MSF_sc_ per city was plotted against the timing of sunrise and sunset for the day the survey was completed, as well as time spent outside and light dose (Figure 3). The timing of sunset showed a positive association with MSF_sc_: the later sunset, the later MSF_sc_. Interestingly, no association was seen for sunrise. Collectively, these data suggest that the timing of sunset and therefore the amount of light in the evenings may be more influential on sleep midpoint on free days than the amount of light in the morning in this population.

## Discussion

The findings from this study suggest that in a university student population, a later chronotype is associated with (i) living further from the equator, (ii) a later sunset, (iii) spending more time outside and (iv) waking up after sunrise. Significantly, we did not find that light intensity was associated with chronotype. Initially, these findings appear to contradict earlier studies where increased light exposure is associated with an earlier chronotype in the general population (Roenneberg and Merrow 2007; Wright et al. 2013; Roenneberg et al. 2015; Stothard et al. 2017). However, our findings are derived exclusively from a university student population aged between 17 and 26 years. Thus, we suggest that the age and occupation of our population increase the likelihood that these individuals will experience relatively little light exposure in the morning whilst encountering more light exposure later in the day, when light has a delaying effect upon the circadian system.

In a sample of approximately 200,000 individuals from primarily Central Europe and North America, spending more time outside was associated with an earlier chronotype (Roenneberg and Merrow 2007; Roenneberg et al. 2015). However, when age was taken into consideration, 15–20 year olds did not show a significant correlation between time spent outside and chronotype, and 20–25 year olds had only a weak correlation (Roenneberg et al. 2015). Our sample of over 6000 students (17–26 years) falls across these age ranges and also differs from the Roenneberg sample (MCTQ database) in several important aspects. A key difference is the work status of the populations studied: all individuals within our sample are university students. However the general population sample from the MCTQ database would have included individuals who were studying at school or university, or working. It is possible, therefore, that imposed work schedules could result in more morning vs. evening light exposure in the general MCTQ population. In addition, the data for the current study was collected exclusively in May and October, whilst the general population sample was collected all year round, which might also have an impact upon the timing of light exposure.

The timing of light exposure has a differential effect upon circadian phase: early light exposure advances the cycle whilst light late in the internal day delays circadian phase (Czeisler et al. 1989; Khalsa et al. 2003). In our student population, we found that longer time spent outside the later chronotype, which would suggest that our population was exposed to more phase delaying evening light than phase advancing morning light. Although it was not possible to determine the timing of light exposure definitively from our study, we provide several lines of evidence that support the importance of evening light in this population. In the present study, we demonstrated that the later students wake up after sunrise the later MSF_sc_. As a result, individuals are likely to be exposed to a photoperiod with a greater proportion of evening phase delaying vs. morning phase advancing light. Clearly, future studies will need to define the phase relationship between the internal circadian and external environmental light cycle. Moreover the timing of sunset rather than sunrise was found to be most associated with MSF_sc_ in our population. Previously, a longitudinal study of around 55 000 individuals has reported that MSF_sc_ tracks sunrise and not sunset (Kantermann et al. 2007). However, again, the broad demographics of this population make direct comparisons to our population difficult, but it is possible that the association of young adult university students (comparable to our population) is masked by other individuals in the sample. Finally, the sensitivity of young adults to evening light has recently been demonstrated in two studies. In 20 healthy young adults (mean age of 23), later light onset and offset has been associated with later melatonin onset as assessed using dim light melatonin onset (Wams et al. 2017). A mathematical model of sleep timing based on the experimentally derived effects of light on the human circadian clock, and interaction of the circadian clock and sleep homeostat, predicts a similar finding. Individuals with a longer intrinsic clock and hence later chronotype are predicted to be more susceptible to evening light, causing even more of a delay in the circadian cycle (Skeldon et al. 2017).

Geographical location was found to be associated with chronotype: the closer to the equator the earlier chronotype, and in this regard, our findings are consistent with previous findings (Miguel et al. 2014). However, this association was assumed to be driven by higher environmental light intensities closer to the equator. Interestingly, it was only the duration of time spent outside – not the intensity of light – that was found to influence MSF_sc_ in our study except for subjects in Auckland. Although the intensity of light has been shown to impact on the entraining properties of light pulses under experimental conditions (Boivin et al. 1996; Zeitzer et al. 2005; Duffy and Czeisler 2009), a saturation effect on shifting the phase of the melatonin rhythm has been reported above approximately 1000 lux (Zeitzer et al. 2000), equivalent to approximately 7.9 W/m^2^ (based on the approximation that 1 lux = 0.0079 W/m^2^ for solar irradiance). Considering the lowest average light intensity reported in this study was 19.43 W/m^2^, and therefore well above saturation intensities, it is perhaps unsurprising that no effect of light intensity emerged. Instead, in our population, it appears that the association between chronotype and geographical location is due to the timing of sunset.

This study reported on a large sample of over 6 000 university students collected in term time during spring and autumn. Such numbers help mitigate the limitation of a cross-sectional assessment of chronotype. However, longitudinal studies are needed to determine precisely how an individual’s chronotype changes with environmental light levels and age. Although based on self-reported sleep timings, the MCTQ is a validated measure of chronotype (Kantermann et al. 2015). The reliability of self-reported time spent outside as a proxy for light dose is less certain. The amount and type of environmental light exposure will be influenced by various factors including photoperiod, weather conditions and the level of urbanisation. Although these have been taken into consideration in this study as much as possible (weather conditions accounted for in measures of daily irradiance and photoperiod in proportional assessment of daylight students awake for), objective assessment of time spent outside and light monitoring need to be undertaken to define when individuals go outside and the nature of their light exposure (inside vs. outside). Furthermore, exposure to artificial light was not addressed in this study, which of course will have an added impact on circadian physiology. Of particular interest in this young student population is the impact of light-emitting devices on sleep and the circadian clock. Although such devices have been found to impact sleep and circadian timing (Cajochen et al. 2011; Chang et al. 2015), the findings are mixed (Heath et al. 2014; Rangtell et al. 2016) and these changes are often small; thus, the real-world significance of these findings remains unclear (Zeitzer 2015). The findings from this study, however, emphasise that environmental evening light exposure may need to be tailored for different populations. With the rapid growth in the diversity of energy-efficient light-emitting devices, robust, evidence-based advice is needed to ensure that individuals get the right kind of light at the right time of day to reinforce robust entrainment of the sleep–wake cycle.

In conclusion, we report that in this young adult university student population, time spent outside is associated with a later chronotype. This seems to be linked to the fact that this population spends more time outside in the evening and that dusk light exposure will have a phase delaying effect upon their circadian biology. Moreover, we found that the closer students lived to the equator the earlier their chronotype. Significantly, this also appears to be associated with the timing of sunset rather than sunrise. Collectively, our results emphasise the fact that the age and occupation of individuals will likely impact profoundly upon the timing of their light exposure and hence their phase of entrainment. Moreover, this work highlights the need for future longitudinal studies that will define these relationships with greater precision.
