# Supplementary material for: Chronotype and environmental light exposure in a student population
Source: Chronobiol Int. 2018 Jun 18;35(10):1365–74. doi: 10.1080/07420528.2018.1482556 (PMC6234547; doi:10.1080/07420528.2018.1482556)
Supplement: Supplemental Material [file ICBI_A_1482556_SM6531.pdf]

Supplementary materials

Linear mixed-effects model fitting with lme package with R version 3.0.1. All models are levelled against Oxford and, where applicable, females and spring.

Model 1: City/season interaction for MSFsc covarying for age and sex

	Value	Standard error	df	t-value	p-value
Groningen	0.029602	0.10993814	6420	0.389709	0.6968
Munich	-0.713844	0.11259734	6420	0.262900	0.7926
Perth	-0.432622	0.12883189	6420	-5.540898	0.0000
Melbourne	-0.725011	0.22757196	6420	-1.901034	0.0573
Auckland	-0.073771	0.16146449	6420	-4.490223	0.0000
Season	-0.052164	0.14345121	6420	-0.514257	0.6071
Age	0.653705	0.00771388	6420	-6.762391	0.0000
Sex	0.121189	0.03332279	6420	19.617365	0.0000
Groningen:Season	-0.043641	0.15037687	6420	0.805900	0.4203
Munich:Season	0.414374	0.15437806	6420	-0.282688	0.7774
Perth:Season	0.196925	0.24246922	6420	1.708976	0.0875
Melbourne:Season	0.078087	0.25679926	6420	0.766844	0.4432
Auckland:Season	0.029602	0.19952868	6420	0.391358	0.6955

Model 2: City main effect for MSFsc, covarying for age and sex

	Value	Standard error	df	t-value	p-value
Groningen	0.105897	0.07520592	6426	1.40810	0.1592
Munich	0.015766	0.07804303	6426	0.20202	0.8399
Perth	-0.626986	0.09956148	6426	-6.29748	0.0000
Melbourne	-0.282392	0.09693362	6426	-2.91326	0.0036
Auckland	-0.681775	0.09263664	6426	-7.35967	0.0000
Age	-0.051959	0.00769087	6426	-6.75591	0.0000
Sex	0.654364	0.03332293	6426	19.63706	0.0000

Model 3: City main effect of time spent outside

	Value	Standard error	df	t-value	p-value
Groningen	0.2334311	0.08011872	6224	2.913565	0.0036
Munich	0.2844144	0.08214180	6224	3.462480	0.0005
Perth	0.8123195	0.10496695	6224	7.738812	0.0000
Melbourne	-0.0610385	0.10369715	6224	-0.588623	0.5561
Auckland	0.2336802	0.09857029	6224	2.370697	0.0178

Model 4: City main effect of light dose

	Value	Standard error	df	t-value	p-value
Groningen	3.83332	1.086134	6224	3.529326	0.0004
Munich	1.68083	1.113560	6224	1.509421	0.1312
Perth	43.24676	1.422991	6224	30.391460	0.0000
Melbourne	6.72240	1.405776	6224	4.781984	0.0000
Auckland	4.13231	1.336274	6224	3.092416	0.0020

Model 5: City, light dose and time spent outside main effects of MSFsc, covarying for age and sex.

	Value	Standard error	df	t-value	p-value
Groningen	0.100894	0.07551231	6213	1.33613	0.1816
Munich	0.013837	0.07839135	6213	0.17652	0.8599
Perth	-0.558185	0.11545646	6213	-4.83459	0.0000
Melbourne	-0.301662	0.09855992	6213	-3.06069	0.0022
Auckland	-0.677443	0.09315072	6213	-7.27255	0.0000
Age	-0.049472	0.00779893	6213	-6.34338	0.0000
Sex	0.645503	0.03374920	6213	19.12647	0.0000
Light dose	-0.002092	0.00168160	6213	-1.24410	0.2135
Time spent outside	0.047750	0.02282116	6213	2.09236	0.0364

Model 6: City and time spent outside interaction of MSFsc, covarying for age and sex

	Value	Standard error	df	t-value	p-value
Groningen	-0.022591	0.15088398	6209	-0.149725	0.8810
Munich	-0.039096	0.15578814	6209	-0.250954	0.8019
Perth	-0.588174	0.19523150	6209	-3.012700	0.0026
Melbourne	-0.503604	0.17937919	6209	-2.807485	0.0050
Auckland	-0.769867	0.17929166	6209	-4.293935	0.0000
Time spent outside	-0.022882	0.06409468	6209	-0.357007	0.7211
Age	-0.049293	0.00780214	6209	-6.317886	0.0000
Sex	0.644257	0.03378808	6209	19.067591	0.0000
Groningen:Time spent outside	0.060398	0.06649035	6209	0.908370	0.3637
Munich:Time spent outside	0.030927	0.06806064	6209	0.454400	0.6496
Perth:Time spent outside	-0.001014	0.07588791	6209	-0.013359	0.9893
Melbourne:Time spent outside	0.097147	0.07802323	6209	1.245106	0.2131
Auckland:Time spent outside	0.045983	0.07601675	6209	0.604910	0.5453
